# Corrosion-resistant systems of formate packer fluid for G3/N80/TP110SS pipes at high temperature, high pressure and high H_2_S/CO_2_ ratios

**DOI:** 10.1098/rsos.180405

**Published:** 2018-07-25

**Authors:** Peng Xu, Zhengwu Tao, Zhihong Wang

**Affiliations:** 1 College of Petroleum Engineering, Yangtze University, Wuhan, People's Republic of China; 2 Hubei Cooperative Innovation Center of Unconventional Oil and Gas, Yangtze University, Wuhan, People's Republic of China; 3 Research Institute of Exploration and Development, Tarim Oilfield Company, PetroChina, Korla, People's Republic of China; 4 Petroleum Engineering & Technology Research Institute, North China oil and gas branch, Sinopec, Zhengzhou, People's Republic of China

**Keywords:** corrosion, high acid gas, formate packer fluid, H_2_S/CO_2_, alloy G3/N80 Steel/TP110SS steel

## Abstract

A series of corrosion problems caused by high-temperature, high-pressure and high-acid gas environments has been an issue in oil and gas production for a long time. During the development of a high-acid gas field, the petroleum pipe is subjected to many aspects of corrosion, and the corrosion mechanism is complicated by the condition of the coexistence of H_2_S/CO_2_. Based on the study of the corrosion problem associated with the formate packer fluid in Southwest China, three kinds of steels were studied for corrosion prevention in the alloy G3/N80 steel/TP110SS steel. The study shows that the corrosion rate of the formate packer fluid is low, but corrosion is severe in environments characterized by high temperatures, high pressures and high-acid gas contents. Based on the consideration of cost and the difficulty of realization, an anti-corrosion system was constructed based on the existing packer fluid, mainly through the introduction of a variety of anti-corrosion additives. Through the selection of various additives and corrosion experiments, a corrosion protection system of formate packer fluid was developed. Corrosion tests show that the corrosion rate of the system must be less than 0.076 mm yr^−1^ to achieve the purpose of corrosion protection. The formate packer fluid with corrosion protection can meet the needs of the current application.

## 1. Introduction

Currently, high acid gases such as in Puguang and Yuanba in northeastern Sichuan are undergoing large-scale development (table 1). Such gas fields are characterized by high temperature, high pressure, high CO_2_ content and high H_2_S content [1,2]. Under the conditions of high temperature, high pressure, high CO_2_ and high H_2_S, the gas field corrosion environment is bad. Tubing, casing and downhole tools are vulnerable to the acid medium corrosion, especially CO_2_ and H_2_S corrosion. Packer fluid is an effective measure to reduce corrosion [3,4]. Packer fluid, which has various properties, such as reducing the pressure difference between the tubing and annulus, preventing packer leakage, reducing oil and gas pressure supported by a casing head or packer, controlling the tubing and casing corrosion, and preventing fouling, is filled between the tubing and casing [5,6]. Conventional packer fluid mainly includes oil-based and water-based fluids. Compared to the problems of the high cost and serious pollution of oil-based packer fluid, a clean brine system has serious corrosion problems, but formate packer fluid has the characteristics of a non-solid and low corrosion itself. Its density falls within a wide adjustment range. The formate fluid does not generate sediment and has low formation damage [7–10]. The non-corrosive characteristics of formate itself further illustrate the degree of corrosion associated with formate packer fluid.

**Table 1.** Nomenclature.

1 g m^−2^ h^−1^	equal to 0.91 mm yr^−1^
1227	benzalkonium chloride, non-oxidative bactericide
7019	RCONH_2_, aliphatic acid amine compound
AV	apparent viscosity, mPa s
BA	number of bacterial colonies in blank sample multiplied by the dilution times
BB	number of survival bacterial colonies after test multiplied by the dilution times
BE	sterilization efficiency, %
C2/C4/C15	imidazolines
CH-5	quaternary ammonium salt cationic polymer bactericide
ClO_2_	chlorine dioxide
CR	sample corrosion rate, mm yr^−1^
CS-30	new compound bactericide
CS	formulation with imidazoline derivatives, thioesters, alkynes, surfactants, organic solvents and other amide compounds
CT10-3	organic guanidine + quaternary ammonium salt + surfactant + solvent
*D*	sample material density, g cm^−3^
DC	oxygen content before addition of deoxidizer
DD	oxygen content after addition of deoxidizer
DR	deoxidization rate
J12	1227 + double oxide + other
KCOOH	potassium formate
KS-1	isothiazolinone compound
KWJ	high-temperature stabilizer
NaCOOH	sodium formate
NY-875	phenol + organic amine + formaldehyde
PAC-HV	high viscous polyanionic cellulose
PAC-LV	low viscous polyanionic cellulose
PV	plastic viscosity, mPa s
QS-1	quaternary ammonium salt cationic polymer bactericide
S15	dithiocyan-based methane + solvent + surfactant
SA	superficial area of sample, cm^2^
SQ8	dithiocyan-based methane + 1227 + solvent + surfactant
*T*	experimental time, h
TY408	oxidative bactericide
TY416	non-oxidative bactericide
*W*_1_	weight before experiment, g
*W*_2_	weight after experiment, g
WC-3	dithiocyanyl methane + disulfone + solvent
WC-85	dithiocyanyl methane + disulfone + solvent
XC	biopolymer
YP	yield point, Pa
YS-02	new quaternary ammonium salt bactericide
ZYJ	new compound bactericide

Although the corrosion associated with formate packer fluid is relatively small, in an environment at high temperature, high pressure and high acid gas, the bad sealing of packer fluid will lead to a large number of unfavourable factors, resulting in high amounts of acid gas entering the packer fluid. CO_2_ and H_2_S reduce the pH value of the packer fluid, resulting in many problems such as an increasing degree of corrosion and formate decomposition [11,12]. CO_2_ or H_2_S corrosion is affected by the gas partial pressure, temperature, water salinity, Cl^−^ content, pH value, etc. The influence of these factors causes the difficulty of protection from CO_2_ or H_2_S corrosion to be relatively large. The corrosion mechanism under the condition of CO_2_ and H_2_S coexistence is more complex [13–15].

According to the current problems of the corrosion of tubing and casing, in most cases, the solution is the main pipe replacement with a pipe that shows good anti-corrosion performance, which solves the corrosion problem to a large extent but also simplifies later work. However, the application of an anti-corrosion pipe column will greatly increase the engineering cost [16–18]. Based on the current treatment methods in the petroleum industry, adjusting the corrosion resistance of the corrosion medium is the main solution to this problem, and this solution can efficiently and simply solve the corrosion problem.

The evaluation and optimization of formate packer fluid was mainly based on the corrosion characteristics of the base fluid of the existing formate packer fluid under the condition of high temperature, high pressure and high acid gas to analyse the corrosion mechanism and optimization mechanism. Based on these mechanisms, this study introduces a corresponding adjusted treatment, evaluates the corrosion effect under different conditions, and constructs a formate packer fluid system that can meet the user requirements under the conditions of high temperature, high pressure, high CO_2_ and high H_2_S content.

## 2. Experimental materials and methods

### 2.1. Experimental materials

#### 2.1.1. Base fluid of formate packer fluid system

The base fluid comprises water + 0.2–0.5 wt% XC + 0.2–0.5 wt% PAC-HV + 0.2–0.5 wt% PAC-LV + 3–5 wt% KWJ + NaCOOH + KCOOH + others. The base performance of a base fluid with 1.30 g cm^−3^ is shown as an example in table 2.

**Table 2.** Base fluid performance of formate packer fluid.

condition	AV (mPa s)	PV (mPa s)	YP (Pa)
before ageing	48	37	11
after ageing at 140°C×16 h	46	40	6

#### 2.1.2. Materials of corrosive pipes

Based on the material selection requirements of tubing and casing in the Puguang acid gas field, three kinds of pipes of alloy G3, N80 steel and TP110SS steel are widely used in drilling engineering. Therefore, the study selects these three types of pipes to carry out the experiments, and the components are shown in table 3.

**Table 3.** Pipe components (wt%).

pipe	C	Si	Mn	P	S	Cr	Ni	Mo	Cu
N80	0.24	0.22	1.19	0.013	0.004	0.036	0.028	0.021	0.019
TP110SS	0.27	0.26	0.60	0.015	0.005	0.600	0.250	0.721	−
G3	0.014	0.62	0.51	0.005	0.001	22.13	45.01	7.130	1.67

### 2.2. Experimental apparatus and methods

#### 2.2.1. Corrosion experiment

The corrosion detection methods are carried out by mechanical methods, including surface inspection, the hanging piece method, etc. Surface inspection uses digital cameras to record the appearance of samples, the changing conditions of the environment and the status of the corrosion products. The hanging piece method involves fixing the sample holder in a high-temperature and high-pressure reactor, observing the surface and measuring the weight loss after a certain period of the operation of the corrosion process.

The hanging piece method and surface inspection were used to measure the corrosion rate for CO_2_/H_2_S under the conditions of different types of steel and different packer fluids. The method for calculating the corrosion rate was formula (2.1).

2.1 $$\mathrm{CR}=\frac{(W_{1}-W_{2})\times 87\;600}{SA\cdot T\cdot D}.$$

The experiment followed the China national standard ‘GB 10124-88 metals materials – Uniform corrosion – Methods of laboratory immersion testing' and the China petroleum industry standard ‘SY/T 5329-2012 reservoir water quality index and analysis method'.

#### 2.2.2. Rheology test

A six-speed rotational viscometer was used to measure the readings under different rotating speeds: $\varphi_{600}$, $\varphi_{300}$, $\varphi_{200}$, $\varphi_{100}$, $\varphi_{6}$, $\varphi_{3}$. Based on formulae (2.2)–(2.4), the parameters of the apparent viscosity (AV), plastic viscosity (PV) and yield point (YP) of packer fluid were calculated.

2.2 $$\begin{array}{rl} \mu_{\mathrm{AV}} & =0.5\varphi_{600}, \end{array}$$

2.3 $$\begin{array}{rl} \mu_{\mathrm{PV}} & =\varphi_{600}-\varphi_{300} \end{array}$$

2.4 $$\begin{array}{rl} \;\text{and}\;\mathrm{YP} & =\mu_{\mathrm{AV}}-\mu_{\mathrm{PV}}. \end{array}$$

#### 2.2.3. Bactericidal efficiency

To test bactericidal efficiency with different concentrations of bactericide on the bacterial colony, an experiment was carried out that used the basic fluid of formate packer fluid for suspending bacteria, using the percentage that represented sterilization (BE) and formula (2.5).

2.5 $$\mathrm{BE}=\frac{\left(\mathrm{BA}-\mathrm{BB}\right)}{\mathrm{BA}}\times 100\%.$$

#### 2.2.4. Deoxidization rate

To test the deoxidization efficiency with different concentrations of deoxidizer, a test was carried out that used the basic fluid of the formate packer fluid as a test object, and the oxygen content tester was used to perform the test, using a percentage representing the deoxidization rate (DR) and formula (2.6).

2.6 $$\mathrm{DR}=\frac{\left(\mathrm{DC}-\mathrm{DD}\right)}{\mathrm{DC}}\times 100\%.$$

## 3. Corrosion mechanism of H_2_S/CO_2_

### 3.1. H_2_S

H_2_S corrosion is caused by the invasion of H_2_S in the formation if the packer has poor sealing or even damage; it may also be produced by the decomposition of a sulfur agent in the formulation of packer fluid under the annulus condition of high temperature and high pressure, such as the decomposition of sulfur-containing corrosion inhibitors [19,20]. H_2_S corrosion used to be called acid corrosion in the oil and gas industry. The H_2_S gas itself usually has no corrosive effect and produces the corrosion of tubing and casing only when H_2_S and water exist simultaneously. In the annulus of the tubing and the casing, the gas containing H_2_S is invaded by the packer fluid into the water-based packer fluid. H_2_S turns into wet H_2_S and then causes corrosion. After the H_2_S is dissolved in the packer fluid solution, the dissolved H_2_S is ionized rapidly, and the dissociation reaction is shown below [21].

$$\begin{array}{r} {\text{H}}_{2}\text{S}\;\text{(aq})\to {\text{H}}^{+}\text{(aq})+\text{H}{\text{S}}^{-}(\text{aq})\\ \;\text{and}\;\text{H}{\text{S}}^{-}(\text{aq) }\to {\text{H}}^{+}(\text{aq) }+{\text{S}}^{2-}(\text{aq}). \end{array}$$

H^+^ is a strong depolarizing agent that takes electrons from the surface of the tubing and casing and then restores itself to become hydrogen atoms. This process is called the cathodic reaction. When the main components of the tubing and casing steel lose electrons, the steel becomes Fe^2+^. Fe^2 +^ and S^2 –^ can react to form FeS, and this process is called the anode reaction. The above electrochemical reactions are often expressed as follows:

$$\begin{array}{rl} & \text{Fe}\to \text{F}{\text{e}}^{2+}+2\text{e}\;(\text{anodic reaction) , }\\ & 2{\text{H}}^{+}+2\text{e}\to {\text{H}}_{2}\;(\text{cathodic reaction}),\\ & \text{F}{\text{e}}^{2+}+{\text{S}}^{2-}\to \text{FeS (anodic product) }\\ \;\text{and}\; & \text{Fe}+{\text{H}}_{2}\text{S}\to \text{FeS}+{\text{H}}_{2}\;(\text{total electrochemical reaction)}. \end{array}$$

These reactions produce large amounts of hydrogen atoms and provide the material conditions for hydrogen embrittlement. The large amounts of H_2_S (aq) and HS^−^ in the packer fluid can inhibit the reaction of hydrogen atoms (H) to produce hydrogen molecules (H_2_). When excess hydrogen is contained, a certain hydrogen pressure is formed, causing the hydrogen to penetrate and enrich the defects in the steel [21]. The deposition of the anode product FeS on the surface of the tubing and casing steel can form a denser passivation film to prevent further corrosion. Then, in the environment of coexisting CO_2_, O_2_ and Cl^−^, the passivation film is vulnerable to damage and cannot prevent further corrosion.

### 3.2. CO_2_

When CO_2_ is dissolved in packer fluid, it has a strong corrosive effect on steel in the process of operation, and the total acidity is higher than that of hydrochloric acid at the same pH value. The most typical characteristics of CO_2_ corrosion are shown as local pitting corrosion, outline corrosion and large area pitting corrosion. Large area pitting corrosion reflects the most severe corrosion. The corrosion perforation rate is very high. The corrosion rate reaches up to 3–7 and up to 20 mm yr^−1^ under anaerobic conditions, making the tube life fall fast. The corrosion process of CO_2_ is as follows: when the CO_2_ meets the water-based packer fluid, a certain amount of CO_2_ will be dissolved in water to form a solution of a certain CO_2_ concentration. The solubility of CO_2_ in water will depend on the temperature and CO_2_ partial pressure. The CO_2_ dissolved in water reacts with water to form carbonic acid [22]:

$$\text{C}{\text{O}}_{2}+{\text{H}}_{2}\text{O}\to {\text{H}}_{2}\text{C}{\text{O}}_{3}.$$

The first hydrolysis of carbonic acid: ${\text{H}}_{2}\text{C}{\text{O}}_{3}\to {\text{H}}^{+}+\text{HC}{{\text{O}}_{3}}^{-}$. The second hydrolysis of carbonic acid: $\text{HC}{{\text{O}}_{3}}^{-}\to {\text{H}}^{+}+\text{C}{{\text{O}}_{3}}^{2+}$. The reaction of H_2_CO_3_ and Fe in the solution contributes to the corrosion of Fe:

$$\begin{array}{rl} & \text{Fe}\to \text{F}{\text{e}}^{2+}+2\text{e}\;\text{(anode reaction), }\\ & 2{\text{H}}^{+}+2\text{e}\to {\text{H}}_{2}\;\text{(cathodic reaction) }\\ \;\text{and}\; & \text{Fe}+{\text{H}}_{2}\text{C}{\text{O}}_{3}\to \text{FeC}{\text{O}}_{3}+{\text{H}}_{2}\;(\text{total electrochemical reaction}). \end{array}$$

The second step of the hydrolysis is very weak. The solutions mainly exist as H^+^ and HCO^3−^. Most of the substances in the reaction products are Fe (HCO_3_)_2_. Fe (HCO_3_)_2_ is not stable in the high-temperature stratum, and it is easy to break down to form:

$$\text{Fe}{\left(\text{HC}{\text{O}}_{3}\right)}_{2}\to \text{FeC}{\text{O}}_{3}+{\text{H}}_{2}\text{O}+\text{C}.$$

CO_2_ corrosion is a typical local corrosion, with carbonate or film generated by corrosion forming corrosion with a strong autocatalytic effect between different regions. CO_2_ partial corrosion is the result of corrosion galvanic action [23].

### 3.3. Corrosion mechanism with the coexistence of CO_2_ and H_2_S

Most of the gas fields are characterized by high temperature, high pressure, high CO_2_ and high H_2_S, and most corrosion problems and their respective mechanisms have been solved using problem-solving methods. However, the actual situation of the underground gas in the gas fields is the coexistence of CO_2_ and H_2_S. These coexistence conditions cause serious corrosion problems. The study of the corrosion mechanism and the protection methods with the coexistence of CO_2_ and H_2_S must be vigorously carried out [24].

At present, the study of corrosion problems under the condition of the coexistence of CO_2_ and H_2_S is both less and more dispersed, as no systematic and comprehensive in-depth study has been performed. However, domestic and foreign scholars have also done some fruitful work in some respects, and a series of research results has been obtained. The results of corrosion experiments on N80 steel by Zhenquan Bai *et al*. [25] and others [26–28] have shown that the effect of H_2_S is similar to the above results. When *P*_CO_2__/*P*_H_2_S_ = 888 > 200, the presence of H_2_S helped slow the corrosion. The surface of N80 steel has a thick and uniform product film, with high adhesion and low corrosion tendency of steel. In the case of *P*_CO_2__/*P*_H_2_S_ = 7 < 200, the steel surface product film is mainly composed of FeS, and the film has good density and high adhesion. Therefore, the uniform corrosion rate of N80 steel is significantly reduced.

## 4. Corrosion evaluation of formate packer fluid

### 4.1. Evaluation of the effect of organic salt on the corrosion rate

**Figure 1.**
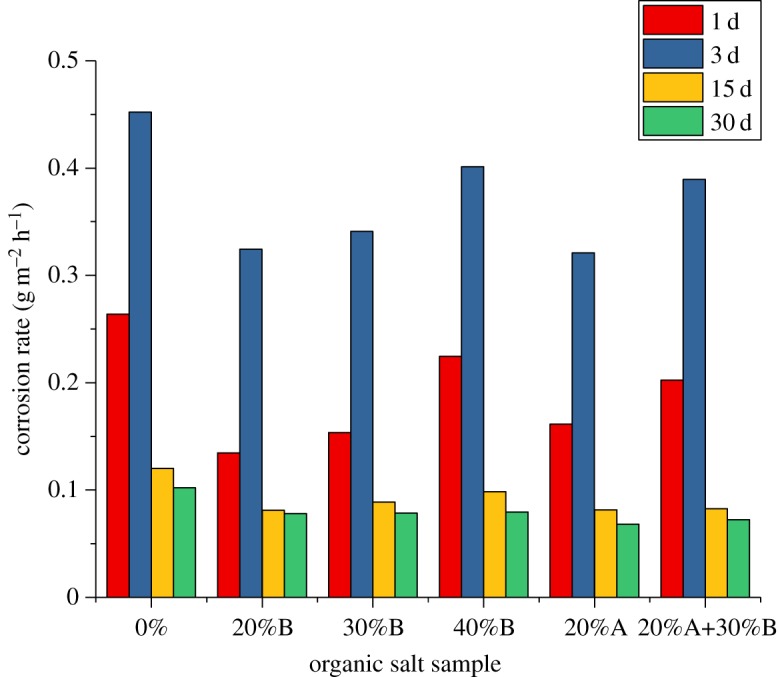
Test on corrosion rate of organic acids at different concentrations (room temperature, N80 steel).

Previous studies have shown that organic salts are much less corrosive to tubing and casing steels than inorganic salts because the product from an organic salt ionization has a neutralizing effect with H_2_S and CO_2_ in the medium. The organic salt also inhibits the corrosion of H_2_S and CO_2_. At the same time, sulfate-reducing bacteria and other biological bacteria have also inhibited growth. Figure 1 is the experimental result of the corrosion rate of an N80 steel sheet with different concentrations of organic salt. Figure 1 shows that the anti-corrosion capacity of formate is limited. Under the influence of long-term acidic medium, to solve the problem of anti-corrosion of casing and other pipes in long-term acidic medium, in addition to using corrosion-resistant materials, adding chemical inhibitors such as corrosion inhibitors into the sealing fluid is a necessary and low-cost method.

### 4.2. Evaluation of corrosion effect in high H_2_S/CO_2_-containing environment

The well annulus of a high-acid gas reservoir is located in an environment characterized by the coexistence of high temperature, high pressure, high H_2_S and high CO_2_. In addition, the corrosion inhibitor has an antiseptic effect on packer fluid. The sulfur-removal agent plays an important role in controlling H_2_S corrosion. To achieve long-term protection, the synergistic action of the sulfur-removal agent and an inhibitor is needed.

The experimental conditions for simulating the actual corrosive environment are shown in table 4. Alloy G3/N80 steel/TP110SS steel was used as the experimental material. The experiment was carried out in a high-temperature and high-pressure corrosion test kettle. Three parallel samples were used for each material [29–31].

**Table 4.** Experimental conditions of high-temperature and high-pressure corrosion.

total pressure (MPa)	CO_2_ partial pressure (MPa)	H_2_S content (ppm)	temperature (°C)	corrosive time (h)	corrosive medium
7	2.5	1000	120	72	packer fluid

The corrosion rates of the three materials are shown in table 5; these were obtained when there was no inhibitor or sulfur-removal agent in the packer liquid. The corrosion rate of alloy G3 is very low, which is lower than the industry standard of 0.076 mm yr^−1^ (China Petroleum and Natural Gas Industry Standard SY/T 5329-2012 Water quality standard and practice for analysis of oilfield injecting waters in clastic reservoirs), so it can be used safely without adding anti-corrosion ingredients. However, the corrosion rates of N80 and TP110SS are, respectively, 0.6642 and 0.6675 mm yr^−1^, which are much higher than the corrosion rate under the conditions of atmospheric pressure and without adding H_2_S and CO_2_. These experiments show that the degree of corrosion is more serious in the oilfield environment. Based on the NACE standard, the degree of corrosion is serious, and it is necessary to carry out the anti-corrosion of the pipes to work safely.

**Table 5.** Corrosion rate of pipe material without corrosion inhibitor and sulfur-removal agent.

pipe material	corrosion rate (mm yr^−1^)	experimental phenomenon
N80	0.6642	After the experiment, the solution was black and the sample has no local corrosion. Besides G3 steel, the remaining steel surface was seriously corroded.
TP110SS	0.6675	
G3	0.0104	

**Figure 2.**
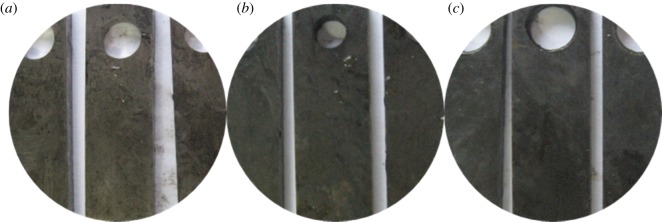
Corrosion morphology of pipes without corrosion inhibitor and sulfur-removal agent. (*a*) G3, (*b*) N80 and (*c*) TP110SS.

**Figure 3.**
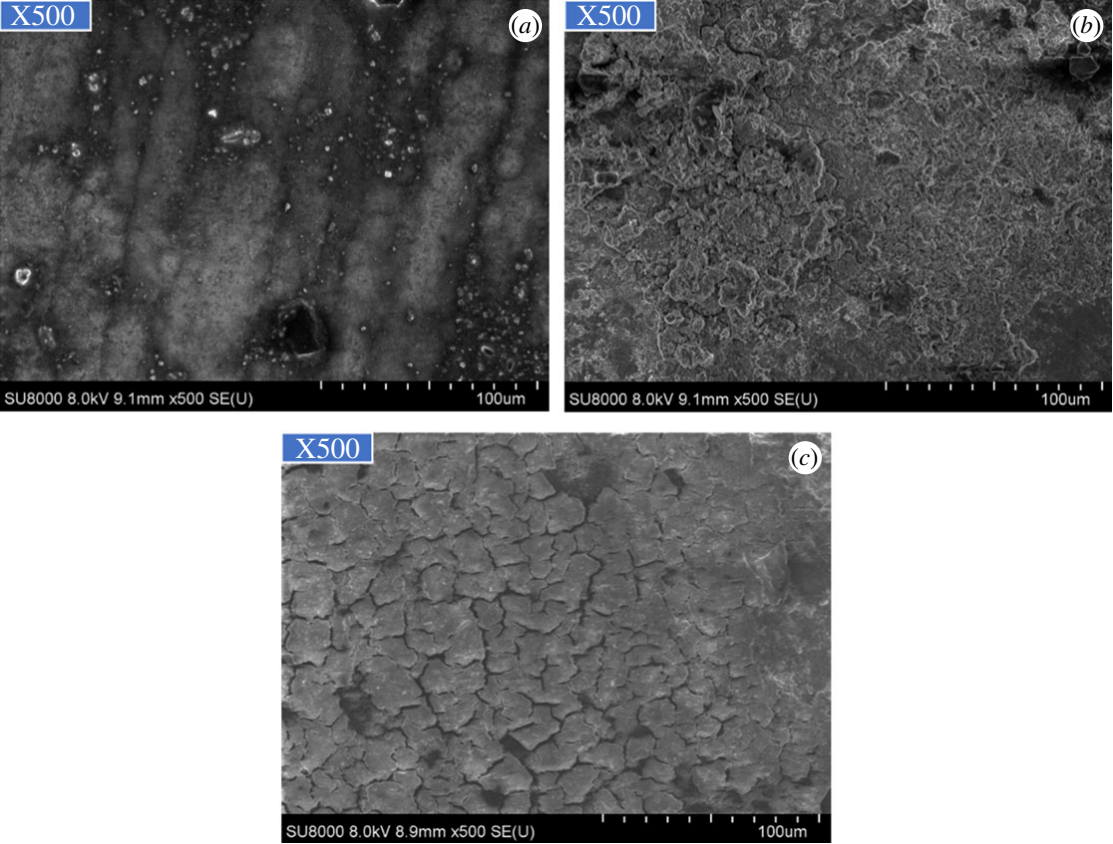
Corrosion microphotographs of pipes without corrosion inhibitor and sulfur-removal agent. (*a*) G3, (*b*) N80 and (*c*) TP110SS.

As figures 2 and 3 show, the corrosion of alloy G3 is very minor, the colour is very shallow, and the colour of the metal can still be seen. The surface of N80 steel and TP110SS steel was completely covered with corrosion product film black, which showed relatively serious corrosion. Corrosion products are found in the removal of firmly attached corrosion products mainly because of the high temperature caused by the crystallization product. This reaction has also been reported in other studies [32,33].

Based on the experimental conditions, we can conclude that H_2_S corrosion should be the main cause for the corrosion of steel in the experimental environment. From the analysis of the H_2_S corrosion mechanism, no matter what form of H_2_S is involved in the corrosion reaction, the adsorption on the steel surface is the basis, and the main factor affecting the adsorption quantity is the content of H_2_S and the number of active points on the steel surface.

In this study, three kinds of samples are adopted. Owing to the large amounts of nickel contained in G3, its corrosion resistance is very high. Previous studies have shown that G3 is a good H_2_S corrosion resistance material. G3 has a low corrosion rate in the H_2_S environment, but the price is more expensive. G3 is generally used only in conditions of a very bad environment. TP110SS is a high sulfur-resistant steel that mainly reflects the ability to resist H_2_S stress corrosion cracking (SCC). However, at high temperatures, SCC has less sensitivity. The main corrosion is weight loss corrosion, and its corrosion rate is larger. The corrosion rate of N80 steel in this environment is similar to that of TP110SS. Therefore, N80 steel can be considered when selecting material, as was also reported in the design of the Puguang Gas Field. N80 steel and corrosion inhibitor could be an anti-corrosion option to put into production.

**Figure 4.**
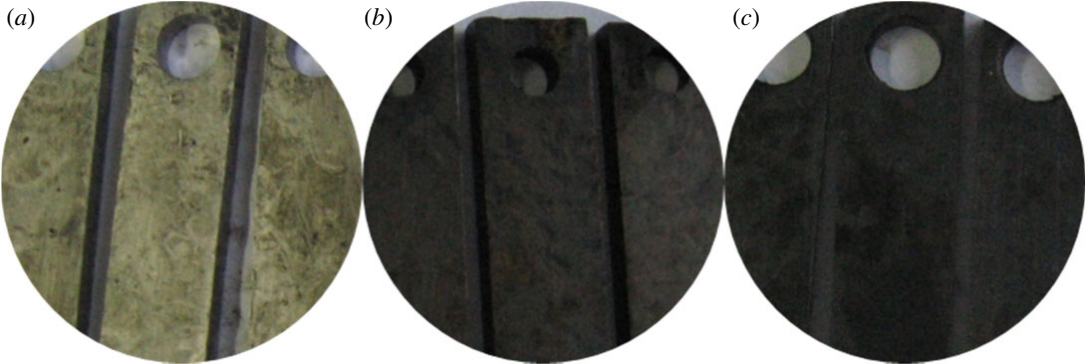
Surface morphology of pipe after removing film. (*a*) G3, (*b*) N80 and (*c*) TP110SS.

Figure 4 shows the surface morphology of the three materials after removing the corrosion products. Pitting corrosion does not occur in the short term.

**Figure 5.**
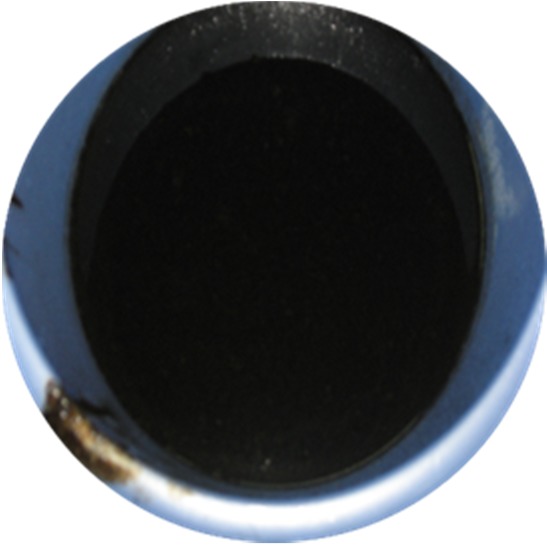
Solution without corrosion inhibitor and sulfur-removal agent after corrosion test.

Before the experiment, the packer fluid was milky white, and the pH value was 8. After the corrosion test, the solution became black (as shown in figure 5), and the pH value changed to 6. The black substance is the corrosion product dissolved in the protective liquid. Because of the high content of H_2_S, the corrosion should be dominated by H_2_S corrosion, and the product is Fe_x_S_y_. Because H_2_S and CO_2_ are dissolved in water, the hydrolysis is acidic, which can neutralize the alkaline packer fluid and decrease the pH value, thus exacerbating the corrosion.

## 5. Construction method of formate packer fluid anti-corrosion system and optimization of treatment agents

### 5.1. Construction method of formate packer fluid anti-corrosion system

In view of the emphasis in the study, the design of the wellbore structure and the selection of materials have been determined. The key point of the research must be focused on the inhibition of the corrosion of the three kinds of tubes in the packer fluid. Corrosion control by chemical method is the most effective and economical method.

Based on the evaluation of the corrosion effect of formate packer fluid under the conventional environment and the high H_2_S/CO_2_ environment, in the case of highly acidic gas, the formate packer fluid system still exhibits serious corrosion. Highly acidic gas corrosion under high-temperature and high-pressure conditions needs to be optimized to meet the requirements of the corrosion performance of packer fluid in oilfield applications.

To solve the corrosion problems in an environment of high temperature, high pressure and highly acidic gas, there are two main methods: replacing the corrosion-resistant steel and improving the anti-corrosion performance of the packer fluid system. In the current environment of the overall downturn in the oil industry, reducing costs is the first problem to be solved. Corrosion-resistant steel will greatly increase the cost of drilling and completion engineering. Corrosion-resistant steel is not completely anti-corrosive, and its anti-corrosion capability needs further tracking and optimization. Improving the corrosion resistance of the packer fluid system is a method that can be easily realized and can also rapidly improve the corrosion resistance. Improving the corrosion resistance of the packer fluid system can reduce the corrosion rate of steel at a lower cost.

Based on the above reasons, the corrosion resistance of the oil casing pipe is reduced by adopting the method of constructing the anti-corrosion performance of the sealing liquid system. The anti-corrosion behaviour of the improved packer fluid is mainly aimed at the characteristics of the steel and the source of corrosion. The main strata studied are characterized by high temperature and high pressure, as well as gas with high H_2_S/CO_2_ content. Therefore, this research focuses on the corrosion prevention of three kinds of steel, alloy G3, N80 steel and TP110SS steel, which are mainly used to reduce the corrosion caused by high levels of H_2_S/CO_2_ gas under the conditions of high temperature, high pressure and high H_2_S/CO_2_ content.

From the existing treatment methods, the existing anti-corrosion system based on the existing blocking liquid system can meet the needs of the field and meet the cost requirements. The construction of a corrosion protection system is mainly achieved by the introduction of anti-corrosion additives, including a sulfur-removal agent, corrosion inhibitor, bactericide, deoxidizer, etc.

### 5.2. Sulfur-removal agent

In this study, the sulfur-removal agent is the H_2_S removal agent. At present, sulfur-removal agents in oil and gas fields can be divided into oxidation type and precipitation type agents [34]. An oxidative sulfur-removal agent oxidizes S^2−^ into elemental S or a higher value material of $\text{S}{{\text{O}}_{4}}^{2-}$, thus acting as a sulfur-removal agent. The representative sulfur-removal agent has ClO_2_, H_2_O_2_, K_2_Cr_2_O_7_ and Ca(ClO_2_)_2_.

Precipitation-type sulfur-removal agents generate an insoluble precipitate through the precipitation reaction of S^2−^ combined with other elements. The commonly used precipitation-type sulfur-removal agent has basic zinc carbonate and basic cupric carbonate. The chemical formula of basic zinc carbonate is Zn_2_(OH)_2_CO_3_, and this material has long been used to remove sulfur in water-based drilling fluid and completion fluid.

$$\text{Z}{\text{n}}_{2}{\left(\text{OH}\right)}_{2}\text{C}{\text{O}}_{3}+2{\text{H}}_{2}\text{S}\to \text{ZnS}↓+3{\text{H}}_{2}\text{O}+\text{C}{\text{O}}_{2}↑.$$

When the solution pH value is between 9 and 11, basic zinc carbonate is undissolved. The sulfur-removal effect is poor. Only when the pH value is higher than 11 is good solubility observed. Because of its solubility limitations, coupled with the fact that Zn^2+^ is a heavy metal ion, we do not select Zn^2+^ as a sulfur-removal agent [35].

**Figure 6.**
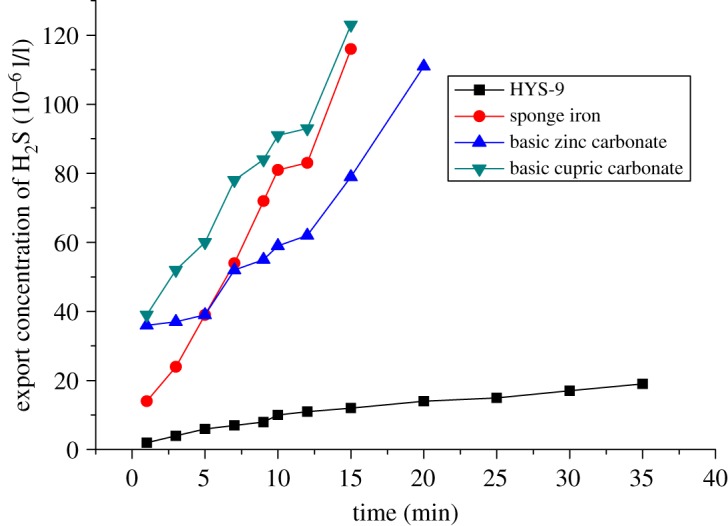
Evaluation of removal efficiency by different sulfur-removal agents.

Based on these data, combined with the actual situation of the Puguang gas field, four kinds of commonly used sulfur-removal agents were evaluated in the laboratory. The four kinds of sulfur-removal agent were HYS-9, sponge iron, zinc carbonate and copper carbonate. HYS-9 is a liquid, and the others are solid. The experiment was carried out by using the normal temperature and atmospheric pressure sulfur-removal tester. In the experiment, the total amount of the four kinds of desulfurizer was 3%, and the sulfur-removal ability was determined by measuring the concentration of H_2_S in the outlet. The experimental data are shown in figure 6.

Figure 6 shows that HYS-9 has the advantages of fast removal of sulfur and high sulfur-removal efficiency. During the compatibility experiment with HYS-9 and the base fluid of packer fluid, no insoluble matter was produced. According to the experimental results, combined with the actual situation, the sulfur-removal agent of HYS-9 was determined as the research system, and the dosage is 1–5 wt%.

### 5.3. Corrosion inhibitor

**Figure 7.**
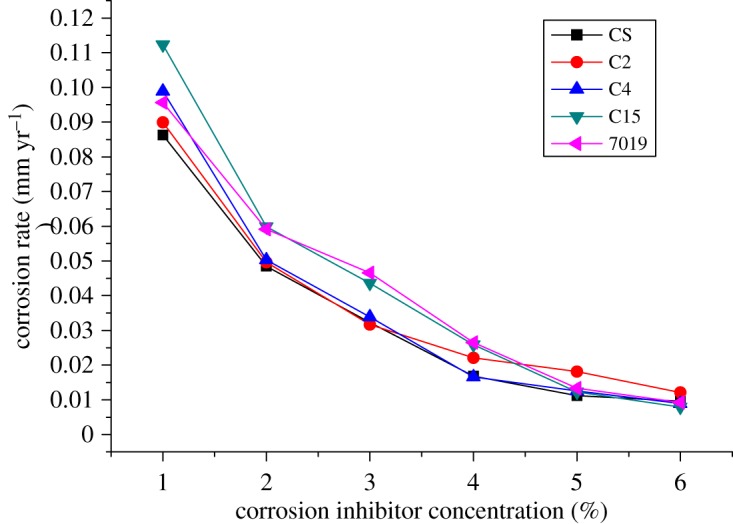
Corrosion rate of N80 steel at different corrosion inhibitor concentration.

During the optimization of the packer fluid, the protective effects of five kinds of corrosion inhibitors, CS, C4, C2, C15 and 7019, were investigated. The inhibitor composition is shown in table 6. Figure 7 shows the corrosion rate of N80 steel with the change in the corrosion inhibitor concentration at room temperature [36]. By comparing the experimental results, all of the corrosion inhibition efficiencies increased with increasing concentration. CS has the best inhibition effect. When a concentration of 5–6 wt% is reached, the corrosion rate changes slowly, and this is the best inhibiting effect.

**Table 6.** Composition information of corrosion inhibitors.

corrosion inhibitor	composition information
CS	formulation with imidazoline derivatives, thioesters, alkynes, surfactants, organic solvents and other amide compounds
C2/C4/C15	imidazolines
7019	RCONH_2_, aliphatic acid amine compound

To further verify the corrosion inhibition effect of the corrosion inhibitor CS, TP110SS steel was used as the experimental material to study its effect, and the amount of CS addition was 5%. The results show that the corrosion rate of TP110SS steel is controlled below 0.001 mm yr^−1^, which shows that the inhibitor CS has a good corrosion inhibition effect on different materials.

**Figure 8.**
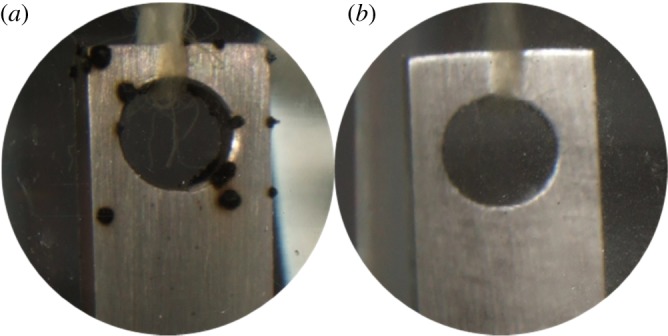
Long-term anti-corrosion effect of CS at room temperature. (*a*) No corrosion inhibitor; (*b*) 3% corrosion inhibitor.

At room temperature, 3 wt% corrosion inhibitor CS was added to the base fluid with a density of 1.27 g cm^−3^, TP110SS steel was placed in solution for 5 months, and its long-term protective performance was measured. The calculated results show that the corrosion rate of the TP110SS steel sheet is 9.5 × 10^−5^/g m^−2^ h^−1^, but the corrosion rate of TP110SS steel sheet is 5.6 × 10^−3^/g m^−2^ h^−1^ without adding the corrosion inhibitor, which shows that the corrosion rate of TP110SS steel is obviously controlled. At the same time, we can observe from figure 8 that pitting corrosion occurs when no inhibitor is added, and pitting pits are not found after adding CS. The above phenomena show that CS has good corrosion inhibition.

### 5.4. Bactericide

The growth, metabolism and propagation of microorganisms in oilfield systems can lead to the corrosion and damage of drilling equipment and other metal materials and the blockage of pipelines [37,38]. As the chemical treatment method is economical, convenient and quick in effect, the chemical treatment has been used as the main treatment method in the actual operation of oilfield production. Bactericides are the most economical and effective way to control bacteria. Bactericides contain substances that can destroy cell enzymes or matrix exchange systems and use their interactions with bacteria to kill or inhibit bacteria. The bactericidal effects of commonly used bactericides and some synthetic bactericides were evaluated and are shown in table 7.

**Table 7.** The detailed data of effect of bactericides.

	bactericidal rate with different concentration				
batericide	20 mg l^−1^	30 mg l^−1^	40 mg l^−1^	60 mg l^−1^	70 mg l^−1^
1227	90	90	90	99	99
CS-30	90	99	99.9	99.9	100
KS-1	90	90	99	99	99.9
YS-02	90	90	99	99	99.9
TY408	90	90	90	99	99
TY416	90	90	90	99	99
SQ8	90	90	90	99	99
S15	90	90	90	99	99
WC-3	90	90	90	99	99
J12	90	90	90	99	99
CT10-3	90	90	90	99.9	99.9
WC-85	90	90	99	100	100
NY-875	90	90	99	100	100
CH-5	90	90	99	100	100
QS-1	90	90	90	99	99
ClO_2_	90	90	90	99	99
ZYJ	90	100	100	100	100

According to the experimental results of table 7, the bactericide has the same bactericidal effects at concentrations of 20 mg l^−1^ and 30 mg l^−1^. When the concentration of the bactericide is 40 mg l^−1^, CS-30 and ZYJ have almost 100% bactericidal effect. When the bacterial concentration is higher, the bactericidal effect of ZYJ is also good. The results of further analyses of the properties of bactericides are shown in table 8.

**Table 8.** Study on the performance of bactericides in the packer fluid.

bactericides	compatibility with organic salt and corrosion inhibitor
CT10-3	no precipitation, no delamination
WC-85	no precipitation, no delamination
NY-875	no precipitation, no delamination
ZYJ	no precipitation, no delamination

According to table 8, the experimental results show that the synthesized bactericide has a high bactericidal rate, good compatibility with organic salts and inhibitors, and no delamination or precipitation in the experiment. Through the evaluation of the above bactericides, ZYJ was identified as a disinfectant for packer fluid.

### 5.5. Deoxidizer

In high-salinity water, corrosion is proportional to the amount of dissolved oxygen in the water. The casing may be perforated by corrosion, resulting in a decrease in casing strength. Therefore, it is necessary to strictly control the dissolved oxygen content in the packer fluid. The main methods adopted at present are physical deoxidization and chemical deoxidization. According to laboratory analysis, the synthetic ZYC is used as the deoxidizer of the packer fluid, and the concentration of the deoxidizer is related to the deoxidization rate, as shown in table 9.

**Table 9.** The detailed data of effect of deoxidizers.

	deoxidization rate with different concentration				
deoxidizer	1%	2%	3%	5%	7%
Rbc	76.1%	82.1%	88.1%	90.1%	90.2%
hydrazine	73.2%	84.1%	88.5%	90.8%	91.0%
Dmko	70.6%	78.6%	84.6%	88.2%	88.2%
AO (acetaldoxime)	78.6%	82.1%	84.6%	87.5%	87.6%
sodium isoascorbate	77.8%	83.4%	86.5%	90.8%	91.6%
*N*-isopropyl hydroxylamine	77.2%	83.5%	88.5%	92.4%	92.6%
methyl ethyl ketoxime	74.6%	81.6%	84.6%	91.0%	92.2%
ZYC	76.4%	90.4%	93.4%	97.8%	98.4%
carbohydrazide (CHZ)	72.8%	82.5%	88.5%	92.8%	93.4%

Table 9 shows that when the concentration of the deoxidizer ZYC reaches 2.0 wt%, ZYC has a high deoxygenation rate of 90.4%, and the experiment determined that the amount of deoxidizer ZYC is 2.0 wt%. Compatibility experiments were carried out. The results showed that ZYC had good compatibility with the formate, inhibitor and bactericide without precipitation or delamination.

## 6. Evaluation of corrosion effect in high-temperature, high-pressure and high-H_2_S/CO_2_ environment

### 6.1. Influence of the sulfur-removal agent

**Figure 9.**
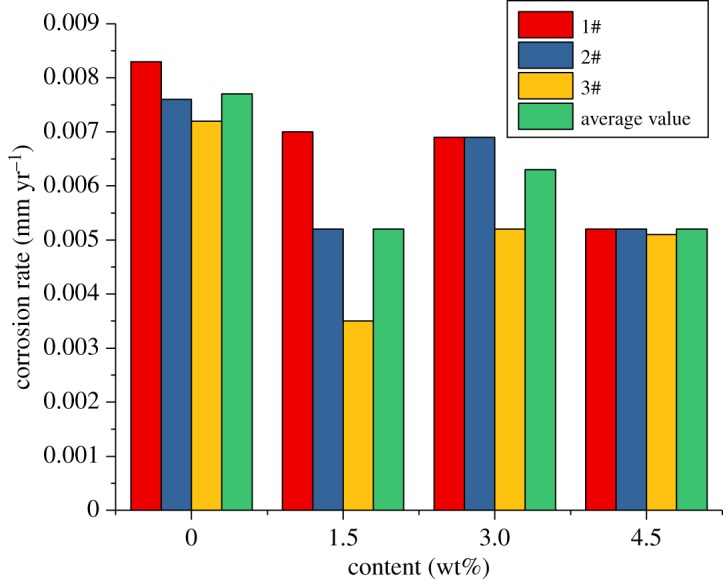
Corrosion rate of alloy G3 (sulfur-removal agent).

**Figure 10.**
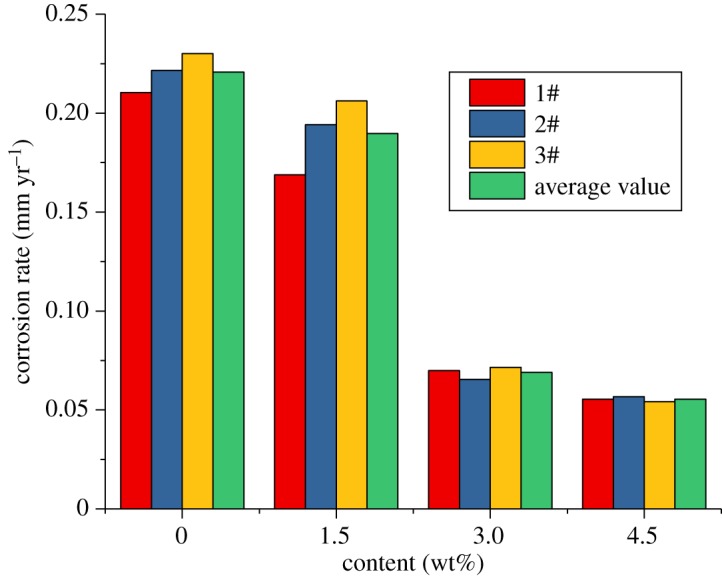
Corrosion rate of N80 steel (sulfur-removal agent).

**Figure 11.**
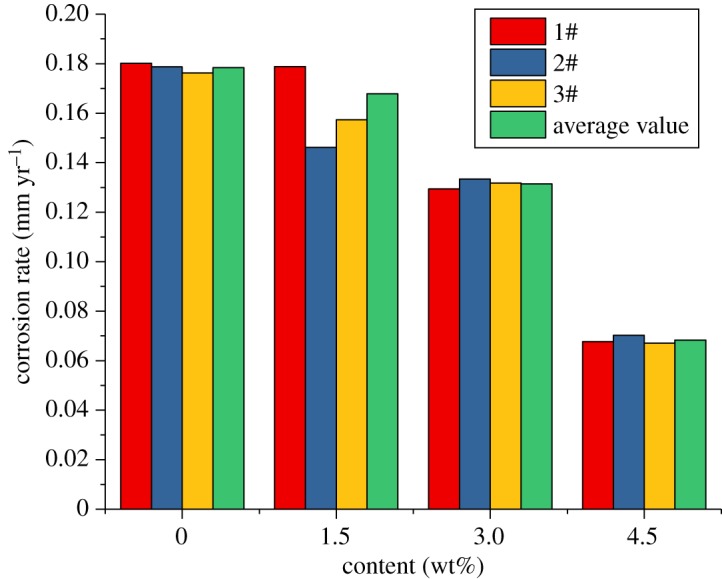
Corrosion rate of TP110SS steel (sulfur-removal agent).

From the previous study, we can see that the corrosion inhibitor CS has the best effect when the concentration is 5–6 wt%. The high-temperature and high-pressure environment is more severe. Therefore, the concentration of 6 wt% was chosen to investigate the influence of the sulfur-removal agent on the anti-corrosion effect. The experimental conditions were the same as those without corrosion inhibitors and sulfur-removal agents. A total of 6 wt% of the inhibitor CS was added to the base fluid of the packer fluid, and 1.5 wt%, 3.0 wt% and 4.5 wt% of the sulfur-removal agent were added. The experimental conditions are shown in table 4. The experimental results are shown in figures 9–11.

For alloy G3, the corrosion rate is very low, but the corrosion inhibitor and sulfur-removal agent can reduce the corrosion rate. The absolute value changes little, and the corrosion rate is much lower than the standard of 0.076 mm yr^−1^. However, the corrosion rates of N80 steel and TP110SS steel sharply decreased with the increase in the concentration of sulfur-removal agent. When the added amount of the sulfur-removal agent was 4.5 wt%, the corrosion rate was less than 0.076 mm yr^−1^. The corrosion was inhibited to a small extent. Compared with no added inhibitor and sulfur-removal agent, the corrosion inhibition efficiency was 91.68% and 89.77%, respectively.

**Figure 12.**
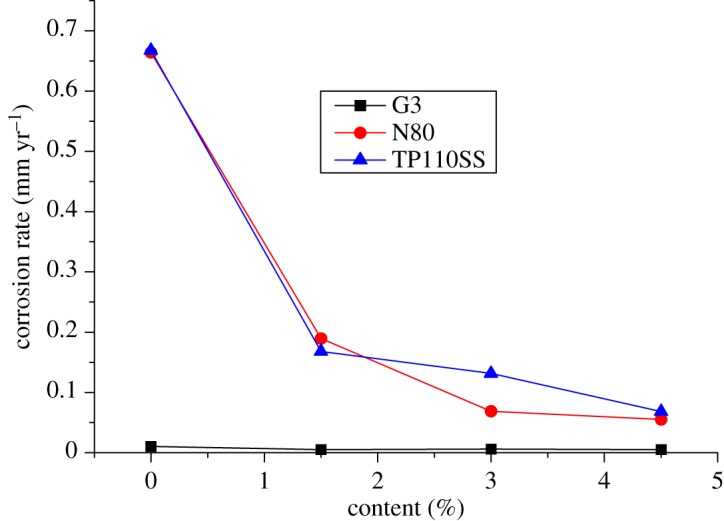
Corrosion rate of pipe varies with the concentration of sulfur-removal agent.

As shown in figure 12, the corrosion rate decreases rapidly when the sulfur-removal agent concentration is low. As the amount of the sulfur-removal agent increased, the decrease in the corrosion rate gradually slowed down. We can predict that the effect of sulfur removal will be increased more slowly by increasing the content of the sulfur-removal agent. The optimum additional amount of the sulfur-removal agent is 4.5 wt%.

**Figure 13.**
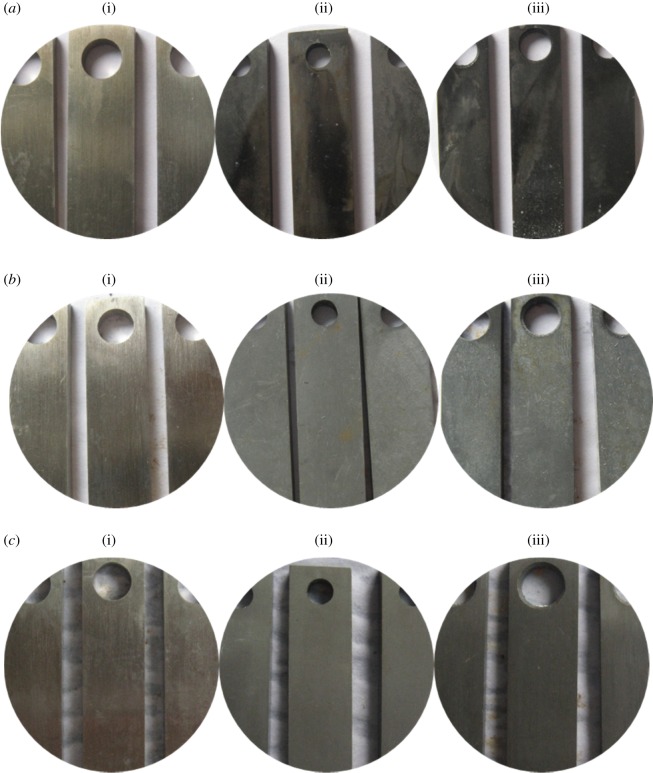
Corrosion morphology of pipe after adding sulfur-removal agent. (*a*) 1.5 wt% Sulfur-removal agent ((i) G3, (ii) N80 and (iii) TP110SS), (*b*) 3.0 wt% sulfur-removal agent ((i) G3, (ii) N80 and (iii) TP110SS), (*c*) 4.5 wt% sulfur-removal agent ((i) G3, (ii) N80 and (iii) TP110SS).

**Figure 14.**
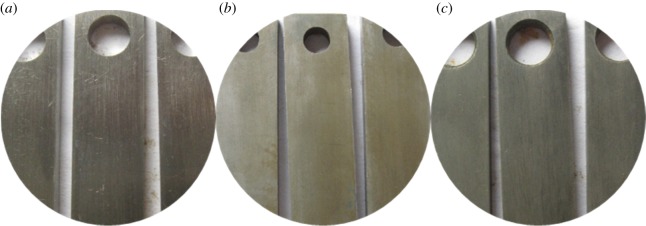
Surface morphology with removing corrosion product after adding sulfur-removal agent. (*a*) G3, (*b*) N80 and (*c*) TP110SS

The corrosion morphology of the tube is shown in figure 13 with the addition of the sulfur-removal agent. For alloy G3, the metal lustre is preserved, and the degree of corrosion is mild. Compared with the corrosion status of steels without adding the sulfur-removal agent and corrosion inhibitor, the corrosion products of TP110SS steel and N80 steel are thinner and more dense after adding the sulfur-removal agent and corrosion inhibitor, and the colour becomes lighter. We can also preliminarily judge that the corrosion rate of the pipe decreases with the increase in the concentration of sulfur-removal agent. After removing the corrosion products, the appearance is shown in figure 14. There is no pitting on the surface of the pipe.

**Figure 15.**
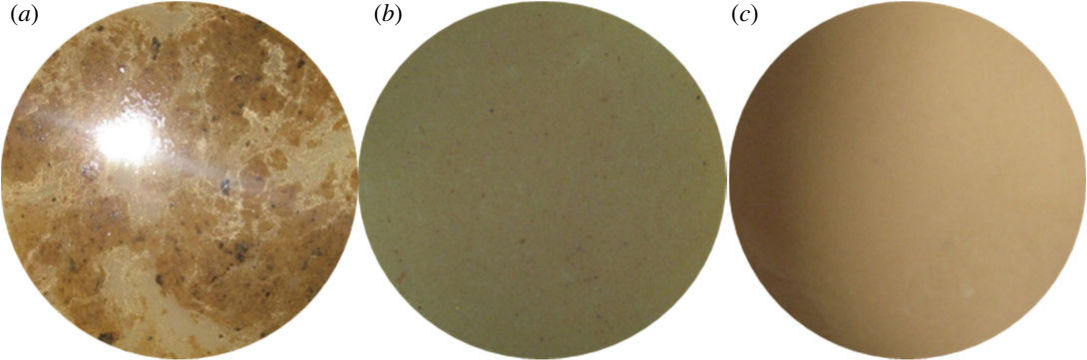
Corrosion test solution after adding sulfur-removal agent. (*a*) 1.5 wt% Sulfur-removal agent, (*b*) 3 wt% sulfur-removal agent, (*c*) 4.5 wt% sulfur-removal agent.

The comparison of the packer fluid properties before and after the corrosion test is shown in table 10. The colour and smell show that the solution is black and has a strong H_2_S smell when no sulfur-removal agent is added. A great change has been made compared to the status without corrosion, which indicated that H_2_S has caused the severe corrosion of the steels. When the sulfur-removal agent is added, the colour becomes milky. With an increase in the content (as shown in figure 15), compared with the corrosion before the test, the effect of removing sulfur becomes better and better, and the corrosion rate of the pipe is also restrained.

**Table 10.** Properties of packer fluid before and after corrosion test.

amount of sulfur-removal agent	pH value before test	pH value after test	solution colour before corrosion	solution colour after corrosion	solution odour after corrosion
0	8	6.0	light ivory	black	H_2_S smell
1.5%	9	7.0	milky white	milky white, a lot of corrosion products on the surface	a small amount of H_2_S smell
3.0%	10	7.0	milky white	milky white, little corrosion products on the surface	no obvious H_2_S smell
4.5%	10	7.0	milky white	milky white	no obvious H_2_S smell

Before adding the sulfur-removal agent, the pH value of the solution is 9. After adding the sulfur-removal agent, the pH value of the solution increases to 10, mainly because the sulfur-removal agent is alkaline. After corrosion, the pH value of the solution changes to 7 because of the reaction between the H_2_S dissolved in the solution and the sulfur-removal agent. Meanwhile, dissolved CO_2_ also lowers the pH value. For general acid corrosion, the pH value is 4–5, and under this formula, the pH value is neutral, and the corrosion is suppressed to a great extent. Therefore, the corrosion rate is much lower than that of formation water containing H_2_S in the literature.

The main function of the sulfur-removal agent is to reduce the effective concentration of hydrogen sulphide so that the chance of the hydrogen sulphide contacting the pipe surface decreases and the corrosion rate is reduced. According to the corrosion mechanism, a decrease in the corrosion rate is realized because the cathode and anode of the corrosion reaction are inhibited, the adsorption capacity of the anode hydrogen sulphide is reduced, and the cathode hydrogen ion cannot participate in the reaction, so the resistance to corrosion is greatly increased.

### 6.2. Influence of corrosion inhibitor

**Figure 16.**
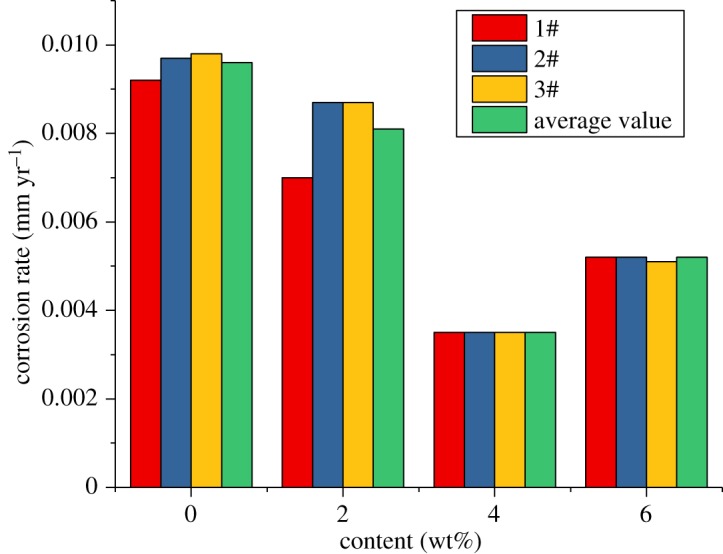
Corrosion rate of alloy G3 (corrosion inhibitor).

**Figure 17.**
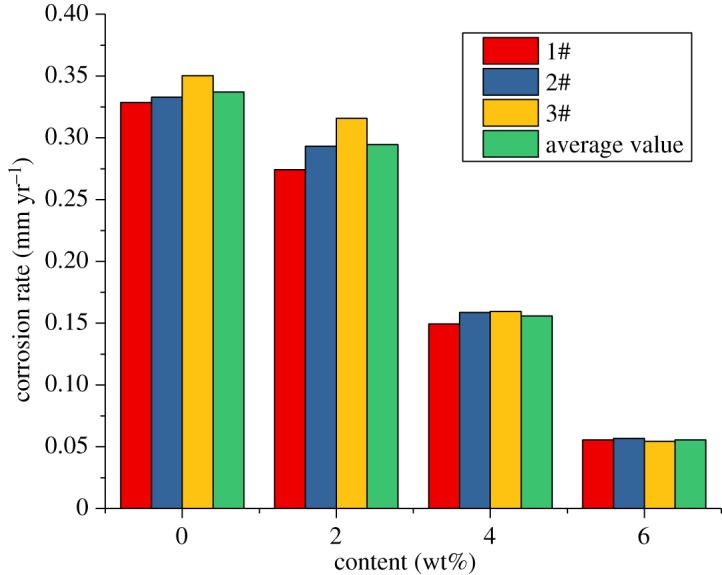
Corrosion rate of N80 steel (corrosion inhibitor).

**Figure 18.**
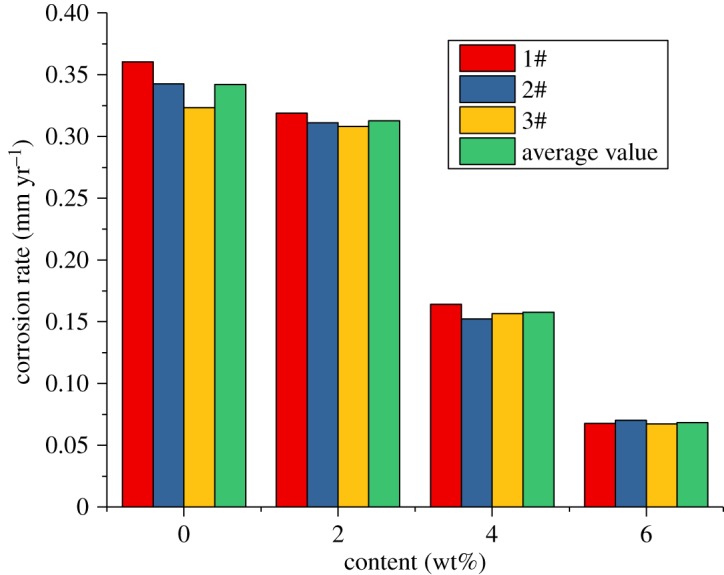
Corrosion rate of TP110SSS steel (corrosion inhibitor).

The results of the sulfur-removal agent show that when the inhibitor content is certain, and the sulfur-removal agent content is 4.5 wt%, the effect is the best. Therefore, in this section, the content of the sulfur-removal agent is fixed at 4.5 wt%. To analyse the effect of changes in the corrosion inhibitor content on the corrosion rate and the experimental conditions and effects of the sulfur-removal agent, the sulfur-removal agent amount in the packer fluid is 4.5 wt% and the inhibitor concentrations are 2, 4 and 6 wt%. The experimental conditions are shown in table 4, and the results are shown in figures 16–18.

**Figure 19.**
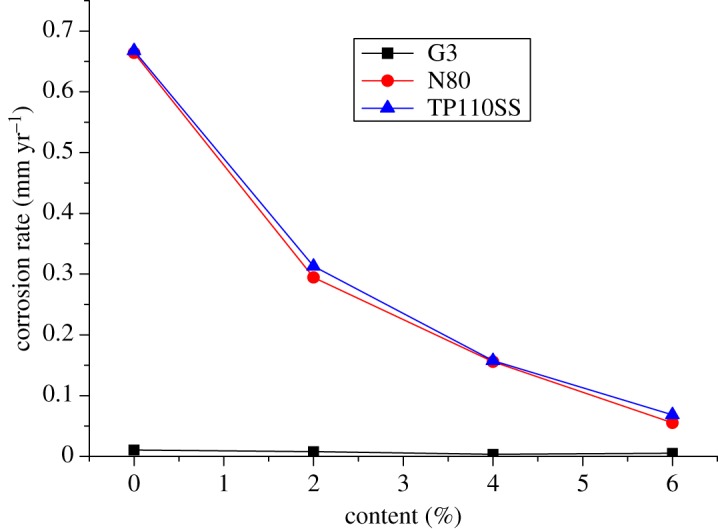
Corrosion rate of steel varies with corrosion inhibitor concentration.

The experimental results show that the corrosion rate of alloy G3 is still far below the standard of 0.076 mm yr^−1^. The corrosion rates of N80 steel and TP110SS steel decrease significantly with the increase in the corrosion inhibitor concentration, and when the amount of sulfur-removal agent is 6 wt%, the corrosion rate is less than 0.076 mm yr^−1^, and the corrosion is suppressed to a very small extent. Compared with the addition of corrosion inhibitor and sulfur-removal agent, the corrosion inhibition efficiencies were 91.68% and 89.77%, respectively. However, if the inhibitor concentration is not enough, N80 steel and TP110SS steel still maintain high corrosion rates, even if the addition of 4 wt% of corrosion inhibitors is far from sufficient. As shown in figure 19, when the inhibitor concentration was low, the corrosion rate decreased rapidly. Therefore, the addition of corrosion inhibitors should be more accurate than the sulfur-removal agent in the application. The results of single agent optimization of corrosion inhibitor showed that when the dosage of CS reached 5–6 wt%, the corrosion rate changed slowly, and the corrosion rate was less than 0.01 mm yr^−1^, which is the main basis for the selection of the corrosive dosage. The addition of corrosion inhibitors has reached 6%, from economic and formula maintenance, and it is also necessary to control the amount of additive at the same time. However, the evaluation result of the influence of corrosion inhibitor showed that the corrosion rate of alloy G3/N80 steel/TP110SS steel is less than the China petroleum industrial standard of 0.076 mm yr^−1^ with the packer fluid to which was added 4.5 wt% of sulfur-removal agent and 6.0 wt% corrosion inhibitor. The dosage of corrosion inhibitor has met the corrosion protection requirements of tubing and casing, which do not need to continue to improve the dosage. As a result, the optimum additional amount of the inhibitor is 6 wt%.

The main component of the inhibitor CS is imidazoline, which is an adsorption inhibitor; the imidazoline changes the activation energy barrier of the electrode reaction mechanism by adsorption. This catalytic effect can be applied either to the negative or anodic reactions, or to the cathodic and anodic reactions at the same time, and the change in the activation energy barrier can be increased (negative catalysis). The ring structure of imidazoline contains N atoms, which is the adsorption group, and it is easy to interact with the iron outer nuclear 3D orbit, resulting in stable adsorption. With the increase in the corrosion inhibitor concentration, the adsorption capacity increases. When the inhibitor content is large, the adsorption will tend to reach saturation; then, increasing the amount of corrosion inhibitor will not reduce the corrosion rate. The main function of the inhibitor is to compete with hydrogen sulphide to reduce the adsorption capacity of hydrogen sulphide and thus to reduce the corrosion rate. Therefore, corrosion inhibitors and sulfur-removal agents must be used in coordination to achieve better corrosion resistance.

**Figure 20.**
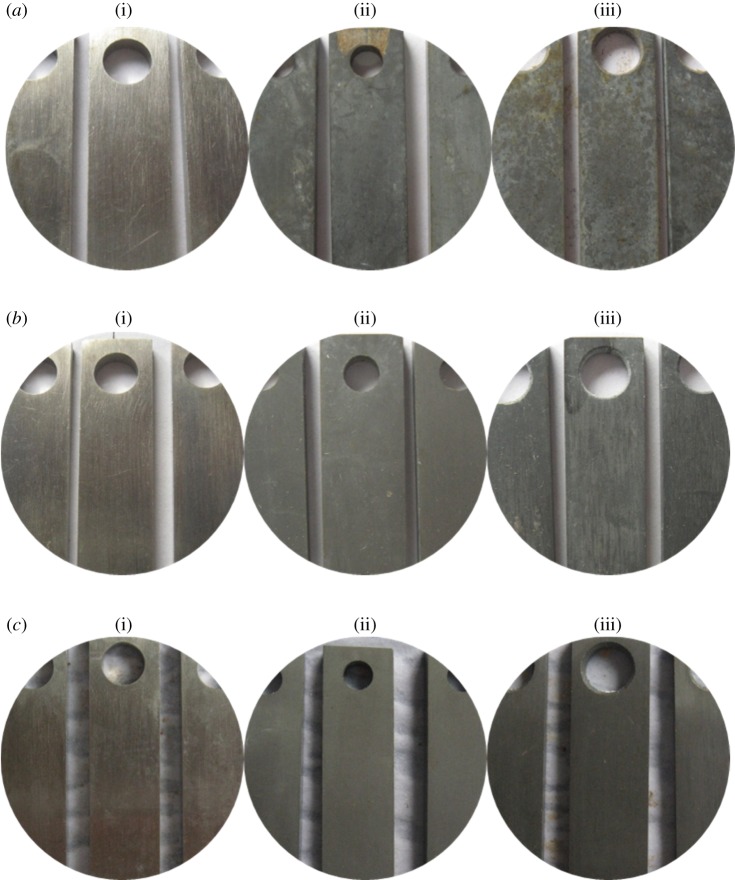
Corrosion morphology of specimens with different corrosion inhibitors. (*a*) 2 wt% Corrosion inhibitor ((i) G3, (ii) N80, (iii) TP110SS), (*b*) 4 wt% corrosion inhibitor ((i) G3, (ii) N80, (iii) TP110SS) and (*c*) 6 wt% corrosion inhibitor ((i) G3, (ii) N80, (iii) TP110SS).

**Figure 21.**
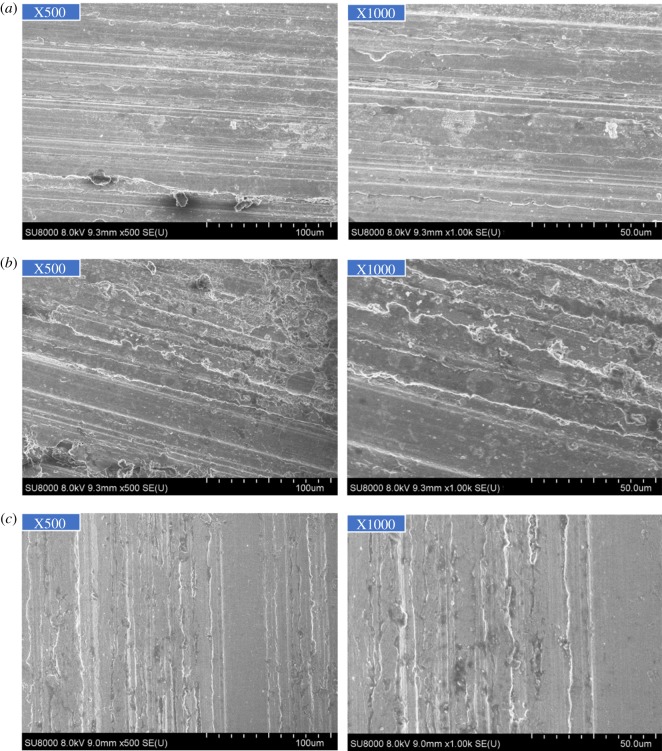
Corrosion microphotographs of pipes with 6 wt% corrosion inhibitor and 4.5  wt% sulfur-removal agent. (*a*) G3, (*b*) N80 and (*c*) TP110SS.

**Figure 22.**
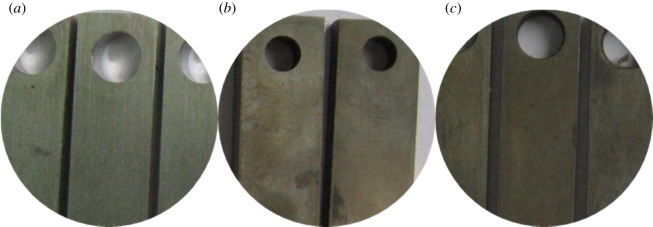
Surface morphology with removing corrosion product after adding corrosion inhibitor. (*a*) G3, (*b*) N80 and (*c*) TP110SS.

After adding different amounts of inhibitor, the corrosion appearance of the pipe is shown in figures 20 and 21. Alloy G3 shows a metallic lustre with slight corrosion. The corrosion products of N80 steel and TP110SS steel are thinner, more compact and lighter in colour than the corrosion products without the addition of sulfur-removal agent and corrosion inhibitor. The morphology indicates that the corrosion rate of the pipe decreases with the increase in the concentration of the corrosion inhibitor. When the amount of inhibitor is 2 wt%, there is a compact surface corrosion product and surface floating layer product on the surface of TP110SS and N80. When the inhibitor content increases, the surface floating layer disappears, and the surface colour changes to grey after corrosion. The morphology after removing the corrosion product is shown in figure 22. Similarly, there is no pitting on the surface of the pipe. As CS is an adsorption inhibitor, pitting corrosion will, in general, not occur.

**Figure 23.**
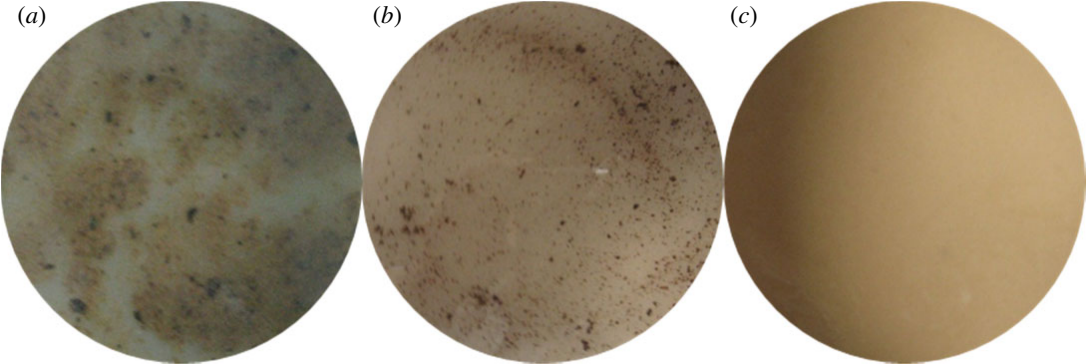
Solution appearance after corrosion tests. (*a*) 2% Corrosion inhibitor, (*b*) 4% corrosion inhibitor and (*c*) 6% corrosion inhibitor.

The comparison of packer fluid properties before and after the corrosion test is shown in table 11. The colour and the smell show that the solution is black and has a strong H_2_S smell when no corrosion inhibitor is added, showing a greatly changed solution compared with no corrosion, indicating that H_2_S has produced severe corrosion on steel. When the corrosion inhibitor is added, the colour becomes milky with an increase in the content (as shown in figure 23). Compared with corrosion before the test, the effect of anti-corrosion is better and better, and the corrosion rate of the pipe is also restrained, similar to the phenomenon observed when adding sulfur-removal agents. Likewise, after adding corrosion inhibitors and sulfur-removal agents, the pH value of the solution becomes 7–7.5, and the corrosion rate decreases.

**Table 11.** Properties of packer fluid before and after corrosion test.

amount of corrosion inhibitor	pH value before test	pH value after test	solution colour before corrosion	solution colour after corrosion	solution odour after corrosion
0	8	6.0	light ivory	black	H_2_S smell
2.0%	9	7.5	milky white	milky white, a lot of corrosion products on the surface	a small amount of H_2_S smell
4.0%	9	7.5	milky white	milky white, little corrosion products on the surface	no obvious H_2_S smell
6.0%	10	7.0	milky white	milky white	no obvious H_2_S smell

### 6.3. Effect of H_2_S content

Since the concentration of downhole H_2_S is variable, the effect of H_2_S content on the corrosion process is investigated by doubling the amount of H_2_S. The experimental conditions are the same as before. The 4.5 wt% of sulfur-removal agent and 6 wt% of corrosion inhibitor are added, and the experimental data for corrosion are shown in table 12. Table 12 shows the corrosion rate of steel is still low with the increase in the H_2_S content, which shows that the packer fluid can be used over a wide range, which is easy to control when used in the field.

**Table 12.** Corrosion rate of pipes when H_2_S content is 2000 ppm.

pipe material	corrosion rate (mm yr^−1^)
N80	0.0604
TP110SS	0.0622
G3	0.0058

**Figure 24.**
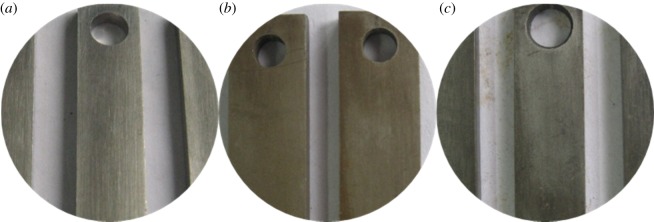
Corrosion morphology at H_2_S content of 2000 ppm. (*a*) G3, (*b*) N80 and (*c*) P110SS.

**Figure 25.**
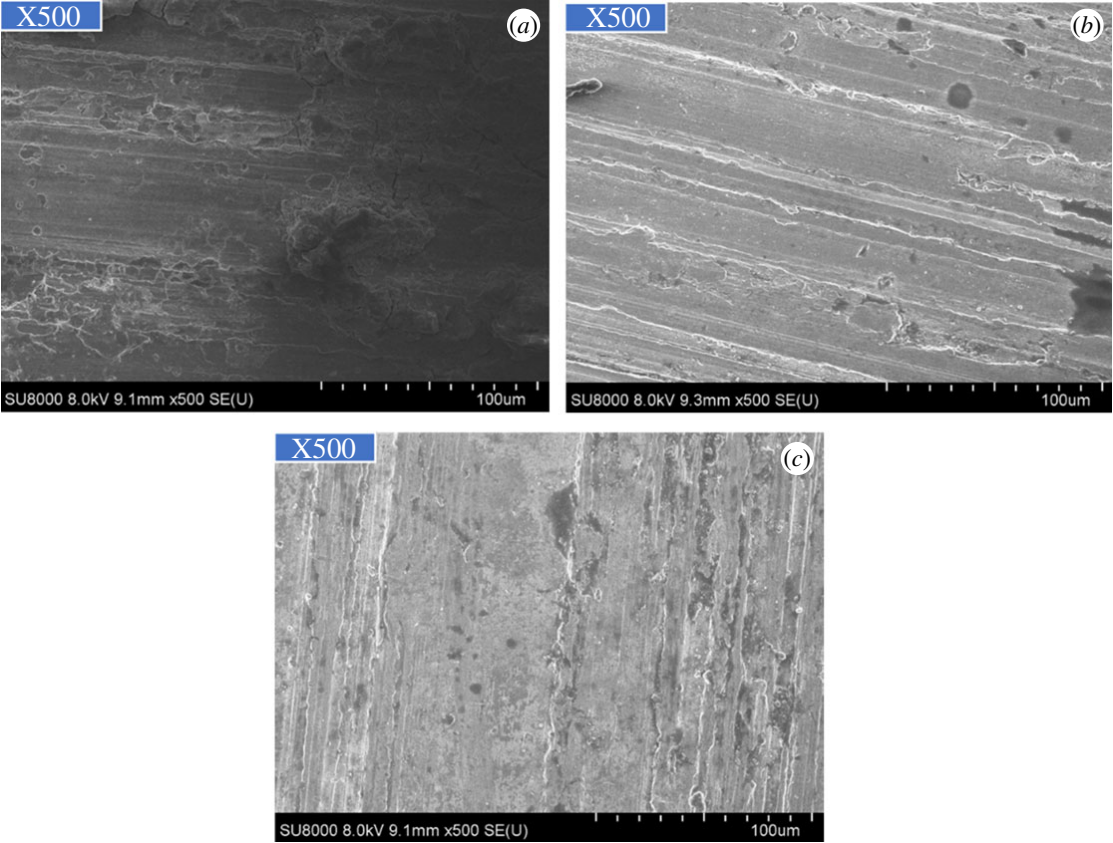
Corrosion microphotographs of pipes at H_2_S content of 2000 ppm. (*a*) G3, (*b*) N80 and (*c*) TP110SS.

**Figure 26.**
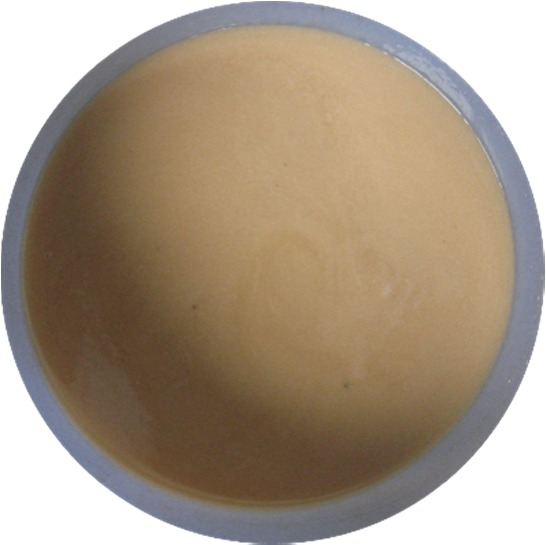
Appearance of corrosion solution at H_2_S content of 2000 ppm.

Figures 24 and 25 show the surface morphology of the sample after corrosion at the H_2_S content of 2000 ppm. The corrosion is very slight, which is similar to that of the H_2_S content of 1000 ppm. Figure 26 shows that this is similar to the H_2_S content of 1000 ppm.

Judging from the above phenomena, we can conclude that when the H_2_S content is doubled, the packer fluid formula is still effective; that is to say, the formula has wide adaptability. The annulus flow of packer fluid will disperse the sulfur-removal agent more evenly. The appropriate velocity will promote the adsorption of corrosion inhibitor and could make a better inhibition effect. Sulfur-removal agent and corrosion inhibitor dosage can be reduced slightly.

### 6.4. Packer fluid after corrosion optimization

The corrosion of alloy G3, TP110SS steel and N80 steel in the base fluid of packer fluid of 1.30 g cm^−3^ density was studied by simulating high temperature and high pressure in an environment containing CO_2_ and H_2_S. The results show that the corrosion rates of TP110SS steel and N80 steel are 0.6668 and 0.6639 mm yr^−1^, respectively, indicating that the corrosion of packer fluid that is filled with CO_2_ and H_2_S is large. Protective measures must be taken, namely, the addition of sulfur and corrosion inhibitor. Adding sulfur-removal agent, corrosion inhibitor, deoxidizer and bactericide is one of the most effective, easy to implement and relatively low-cost measures.

The corrosion of three kinds of steel alloy G3, TP110SS steel and N80 steel in the packer fluid was evaluated after increasing the amount of H_2_S. The results showed that after doubling the H_2_S, the formate packer fluid formula still had good corrosion resistance.

Further simulation experiments of CO_2_ and H_2_S at high temperature and high pressure show that the optimum dosages for the synergistic action of sulfur-removal agent and inhibitor are 4.5 and 6 wt%, respectively. Thus, the final formula for formate packer fluid is obtained: Water + 0.2–0.5 wt% XC+ 0.2–0.5 wt% PAC-HV+ 0.2–0.5 wt% PAC-LV+ 3–5 wt% KWJ + NaCOOH + KCOOH+ 2.0 wt% ZYC+ 4.5 wt% HYS-9 + 0.006 wt% ZYJ + 6.0 wt% CS + others. The range of density regulation is 1.05–1.55 g cm^−3^.

Taking the optimized packer fluid density of 1.30 g cm^−3^ as an example, the rheological properties are shown in table 13.

**Table 13.** The rheological property of optimized packer fluid.

condition	AV (mPa s)	PV (mPa s)	YP (Pa)
before ageing	55.5	44.0	11.0
after ageing at 140°C × 16 h	54.0	46.0	8.0

The data comparison between the two groups of tables 2 and 13 shows that the apparent viscosity and plastic viscosity of packer fluid increase slightly after the addition of sulfur-removal agent, corrosion inhibitor and bactericide, but this change does not affect the use of packer fluid, indicating that the optimized packer fluid meets the design and application requirements in terms of rheological properties.

## 7. Conclusion

(1) High acid gas has serious corrosion effects on the tubing and casing of different steels, and it is necessary to optimize the corrosion of the sealing fluid.
(2) H_2_S/CO_2_/O_2_ can both aggravate pipe corrosion, but under the condition of the coexistence of CO_2_/H_2_S, the corrosion mechanism is more complicated, and the corrosive effect should be considered emphatically.
(3) In response to the corresponding corrosion problems, anti-corrosion additives such as sulfur-removal agent, corrosion inhibitor, bactericide and deoxidizer were introduced, which effectively reduced the corrosion of the packer fluid system to the tubing string.
(4) Under the condition of high temperature, high pressure and high acidity, the effects of different additives and acid gas addition were studied. The results show that the corrosion resistance rate of the sealing liquid is below 0.076 mm yr^−1^ after the addition of anti-corrosion additives, which meets the requirement of pipe corrosion protection.

## Data accessibility

Our data are deposited at Dryad: https://doi.org/10.5061/dryad.p1mf340 [39].
